# Metalloproteomic analysis of liver proteins isolated from broilers fed with different sources and levels of copper and manganese

**DOI:** 10.1038/s41598-024-55478-8

**Published:** 2024-02-28

**Authors:** Renata Aparecida Martins, Andrey Sávio de Almeida Assunção, José Cavalcante Souza Vieira, Leone Campos Rocha, Priscila Michelin Groff Urayama, Marília Afonso Rabelo Buzalaf, José Roberto Sartori, Pedro de Magalhães Padilha

**Affiliations:** 1 School of Veterinary Medicine and Animal Science, São Paulo State University (UNESP), Botucatu, São Paulo Brazil; 2 Institute of Biosciences, São Paulo State University (UNESP), Botucatu, São Paulo Brazil; 3 University of São Paulo (USP), Bauru, São Paulo Brazil

**Keywords:** Biochemistry, Biotechnology, Physiology, Psychology, Chemistry

## Abstract

Supplementing minerals beyond dietary requirements can increase the risk of toxicity and mineral excretion, making the selection of more bioavailable sources crucial. Thus, this work aimed to use metalloproteomics tools to investigate possible alterations in the hepatic proteome of broilers fed with diets containing two sources (sulfate and hydroxychloride) and two levels of copper (15 and 150 ppm) and manganese (80 and 120 ppm), totaling four treatments: low Cu/Mn SO_4_, high Cu/Mn SO_4_, low Cu/Mn (OH)Cl and high Cu/Mn (OH)Cl. The difference in abundance of protein spots and copper and manganese concentrations in liver and protein pellets were analyzed by analysis of variance with significance level of 5%. The Cu and Mn concentrations determined in liver and protein pellets suggested greater bioavailability of hydroxychloride sources. We identified 19 Cu-associated proteins spots, 10 Mn-associated protein spots, and 5 Cu and/or Mn-associated protein spots simultaneously. The analysis also indicated the induction of heat shock proteins and detoxification proteins in broilers fed with high levels of copper and manganese, suggesting the involvement of these proteins in metal tolerance and stress.

## Introduction

Microminerals are indispensable for the growth, health, and performance of animals since they are fundamental in several metabolic processes^1,2^. Copper acts as a cofactor for several important enzymes, such as CuZn-superoxide dismutase (CuZn-SOD), cytochrome-c-oxidase (COX) and ceruloplasmin, which makes it essential in the elimination of free radicals, cellular respiration, and iron mobilization^3^. In the same way, manganese acts as a component of metalloproteins and an enzymatic cofactor, as it is fundamental in antioxidant defense, bone formation and metabolism of carbohydrates, amino acids, and cholesterol^4–6^.

The concentrations of copper and manganese in the ingredients commonly used in broiler feeds are low and usually insufficient to meet nutritional requirements, requiring supplementation to ensure optimal development and productive performance^3,7^. Sources of inorganic minerals such as sulfates and oxides are widely used due to their high commercial availability and low price^2,6^. However, sulfates are known to have low bioavailability due to their high solubility in aqueous media and antagonistic interactions with other minerals and nutrients in the diet. Thus, it is common for the feed industry to adopt a high margin of safety by supplementing levels that exceed nutritional needs, which in turn can harm both animals and the environment due to the risk of toxicity and increased mineral excretion^8^.

Based on this principle, new supplementation strategies that promise greater mineral bioavailability are gaining attention in feed formulation. Among these strategies are hydroxychlorides, also classified as an inorganic source, but with some particularities that differ from sulfates^5^. Hydroxychlorides have strong covalent bonds with minerals, making them less soluble in water and consequently less reactive, thus reducing unwanted interactions with other constituents of the diet^9^. Although these characteristics may theoretically favor the absorption of minerals, studies that explore the functionality of copper and manganese at the molecular level are needed to estimate the bioavailability of different sources more fully.

In this sense, proteomics tools combined with mineral quantification methods can provide relevant information on the incorporation of copper and manganese in proteins, as well as the identification of proteins responsive to different concentrations of these minerals. In addition, metalloproteomics can contribute to the understanding of the mechanisms involved in the toxicity and tolerance of minerals in broilers. Therefore, the objective of this study was to evaluate the differential profile of Cu-associated proteins and Mn-associated proteins in broilers liver proteome fed with two sources (sulfate and hydroxychloride) and two levels of copper (15 and 150 ppm) and manganese (80 and 120 ppm) using metalloproteomic tools.

## Results

The present study used metalloproteomic strategies, combining the evaluation of differential protein expression by two-dimensional polyacrylamide gel electrophoresis (2D-PAGE), the mapping of Cu and Mn in protein spots by graphite furnace atomic absorption spectrometry (GFAAS) and the identification of proteins in spots associated with minerals by liquid chromatography tandem mass spectrometry (LC–MS/MS). Mineral concentrations in liver tissue samples and protein pellets were also evaluated. For the analyzes four treatments were considered: Low Cu/Mn SO_4_ (15 mg kg^−1^ CuSO_4_ and 80 mg kg^−1^ MnSO_4_), High Cu/Mn SO_4_ (150 mg kg^−1^ CuSO_4_ and 120 mg kg^−1^ MnSO_4_), Low Cu/Mn (OH)Cl (15 mg kg^−1^ of Cu(OH)Cl and 80 mg kg^−1^ of Mn(OH)Cl), and High Cu/Mn (OH)Cl (150 mg kg^−1^ of Cu(OH) Cl and 120 mg kg^−1^ of Mn(OH)Cl). Three gels per treatment were considered, and the following comparisons relevant to the study were made: High Cu/Mn SO_4_ x Low Cu/Mn SO_4_; Low Cu/Mn (OH)Cl x Low Cu/Mn SO_4_; High Cu/Mn (OH)Cl x High Cu/Mn SO_4_; and High Cu/Mn (OH)Cl x Low Cu/Mn (OH)Cl.

### Cu and Mn concentrations in hepatic tissue samples and pellets

The copper and manganese concentrations in liver tissue samples and protein pellets are shown in Table 1. According to Table 1, Cu and Mn concentrations in liver tissue and protein pellet were higher in groups high Cu/Mn SO_4_ and high Cu/Mn (OH)Cl compared to groups low Cu/Mn SO_4_ and low Cu/Mn (OH)Cl (*P* < 0.05). In the hepatic tissue, there was no difference in the concentrations of both minerals between the high Cu/Mn SO_4_ and high Cu/Mn (OH)Cl groups and of Mn between the low Cu/Mn SO_4_ and low Cu/Mn (OH)Cl groups (*P* > 0.05). However, the low Cu/Mn (OH)Cl group had a higher concentration of Cu than the low Cu/Mn SO_4_ group (*P* < 0.05). In the protein pellet, the Mn concentration was higher in the low Cu/Mn (OH)Cl group than in the low Cu/Mn SO_4_ group (*P* < 0.05) but did not differ between the high Cu/Mn SO_4_ and high Cu/Mn (OH)Cl groups (*P* > 0.05). The same was observed in the Cu concentrations, except for the high Cu/Mn SO_4_ and high Cu/Mn (OH)Cl groups, where the high Cu/Mn (OH)Cl group had a higher Cu concentration in relation to the high Cu/Mn SO_4_ group.

**Table 1:** Determination of copper and manganese in liver tissue samples and pellets from broiler chickens.

Treatments	Cu concentration (ppm)	Mn concentration (ppm)
Hepatic tissue		
Low Cu/Mn SO_4_	2.60 ± 0.14^c^	10.15 ± 0.07^b^
High Cu/Mn SO_4_	64.30 ± 0.71^a^	64.10 ± 1.56^a^
Low Cu/Mn (OH)Cl	4.80 ± 0.14^b^	10.60 ± 0.28^b^
High Cu/Mn (OH)Cl	71.55 ± 0.35^a^	58.75 ± 1.63^a^
Protein pellet		
Low Cu/Mn SO_4_	2.21 ± 0.03^d^	8.20 ± 0.66^c^
High Cu/Mn SO_4_	55.30 ± 0.95^b^	55.67 ± 0.40^a^
Low Cu/Mn (OH)Cl	4.34 ± 0.05^c^	9.37 ± 0.21^b^
High Cu/Mn (OH)Cl	68.40 ± 0.79^a^	52.60 ± 0.89^a^

^a,b,c,d^ Means followed by different superscript letters in the column differ by Tukey’s test (P < 0.05). Low Cu/Mn SO_4_: 15 ppm Cu sulfate and 80 ppm Mn sulfate; High Cu/Mn SO_4_: 150 ppm Cu sulfate and 120 ppm Mn sulfate; Low Cu/Mn (OH)Cl: 15 ppm Cu hydroxychloride and 80 ppm Mn hydroxychloride; and High Cu/Mn (OH)Cl: 150 ppm Cu hydroxychloride and 120 ppm Mn hydroxychloride.

### Image analysis of gels and mapping of Cu and Mn in spots

**Figure 1:**
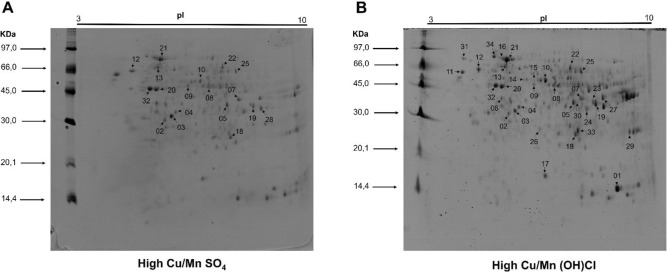
Representation of polyacrylamide gels obtained by 2D-PAGE from liver tissue of broiler chickens from high Cu/Mn SO_4_ (**A**) and high Cu/Mn (OH)Cl (**B**) groups, with associated copper and manganese protein spots indicated with numbers. High Cu/Mn SO_4_: 150 ppm Cu sulfate and 120 ppm Mn sulfate. High Cu/Mn (OH)Cl: 150 ppm Cu hydroxychloride and 120 ppm Mn hydroxychloride. The edges of the gel images were cropped for illustration purposes. See images of uncropped/unedited gels in Supplementary Figure S1 (B) and (D).

The analysis of the digitized gel images obtained in triplicate indicated an average number of 230 ± 69 spots and an average matching of 98 ± 1%, meaning that, on average, 98% of the protein spots were consistently identified and correlated between the different gels analyzed. Representative images of the gels obtained from each treatment are shown in Supplementary Figure S1 [see Supplementary Information]. From GFAAS analysis, 34 differentially expressed spots were associated with copper and/or manganese in the high Cu/Mn SO_4_ and high Cu/Mn (OH)Cl groups. Figure 1 shows the images of the polyacrylamide gels of the high Cu/Mn SO_4_ and high Cu/Mn (OH)Cl treatments with the protein spots associated with copper and manganese indicated with numbers. Nineteen spots were identified with the presence of Cu only, 10 spots with the presence of Mn only and 5 spots with the presence of both minerals. The concentrations of Cu and Mn obtained after acid mineralization of the respective spots are summarized in Table 2. The high Cu/Mn (OH)Cl group had a higher number of spots associated with Cu and/or Mn (33 spots) than the high Cu/Mn SO_4_ group (19 spots).

**Table 2:** Copper and manganese concentrations obtained after acid mineralization of protein spots in the liver tissue of broiler chickens.

Spot ID	Cu concentration (ppb)		Mn concentration (ppb)	
	High Cu/Mn SO_4_	High Cu/Mn (OH)Cl	High Cu/Mn SO_4_	High Cu/Mn (OH)Cl
01		6.81 ± 0.12		
02	3.40 ± 0.07	4.81 ± 0.09		
03	7.30 ± 0.13	9.63 ± 0.15		
04			0.80 ± 0.015	1.19 ± 0.023
05	5.60 ± 0.11	8.39 ± 0.15		
06	4.70 ± 0.08	6.93 ± 0.13		
07	5.40 ± 0.09	9.80 ± 0.17		
08			0.92 ± 0.014	1.80 ± 0.035
09			0.75 ± 0.012	1.50 ± 0.029
10	3.80 ± 0.08	7.21 ± 0.14		
11		7.20 ± 0.14		
12	4.30 ± 0.07	8.04 ± 0.15		
13	3.90 ± 0.08	6.61 ± 0.13		
14		8.75 ± 0.16		1.21 ± 0.024
15		6.63 ± 0.12		1.31 ± 0.026
16				1.28 ± 0.024
17		8.50 ± 0.16		
18	4.10 ± 0.07	7.61 ± 0.14		
19		10.49 ± 0.20	1.15 ± 0.022	2.26 ± 0.044
20	8.50 ± 0.15	11.17 ± 0.21		
21	6.40 ± 0.13	9.63 ± 0.18		
22			0.77 ± 0.014	1.46 ± 0.027
23		8.22 ± 0.14		
24				1.52 ± 0.026
25	3.70 ± 0.06	7.35 ± 0.13	0.74 ± 0.014	1.32 ± 0.025
26				1.35 ± 0.026
27		8.87 ± 0.16		
28	4.50 ± 0.09			
29		7.38 ± 0.14		
30				1.20 ± 0.022
31				1.31 ± 0.025
32	7.10 ± 0.11	11.54 ± 0.22		
33				1.53 ± 0.027
34		9.30 ± 0.17		1.44 ± 0.027

High Cu/Mn SO_4_: 150 ppm Cu sulfate and 120 ppm Mn sulfate; and High Cu/Mn (OH)Cl: 150 ppm Cu hydroxychloride and 120 ppm Mn hydroxychloride.

Differences in the abundance of protein spots associated with Cu and Mn between the groups studied can be seen in Table 3. In the high Cu/Mn SO_4_ group compared to the low Cu/Mn SO_4_ group, 18 spots were differentially abundant. The majority of these spots showed an increase in abundance (13 spots), while 2 were less abundant and 3 were absent. Comparing supplemental levels between hydroxychloride sources (high Cu/Mn (OH)Cl versus low Cu/Mn (OH)Cl), 18 spots also showed differences in abundance. Of these, 10 spots were more abundant, 6 spots were less abundant and 2 spots had a unique presence in the high Cu/Mn (OH)Cl group. When comparing low levels of Cu and Mn between different sources (low Cu/Mn (OH)Cl vs. low Cu/Mn SO_4_), 17 differentially abundant spots were identified, with 8 spots being more abundant, 7 spots being less abundant and 2 spots were absent in the low Cu/Mn (OH)Cl group. In the high Cu/Mn (OH)Cl group compared to high Cu/Mn SO_4_, 18 spots showed differences in abundance, with the majority of these spots being less abundant (12 spots) while 3 were more abundant and 3 had a single presence.

**Table 3:** Relative difference in the abundance of protein spots (t test, *P* < 0.05) associated with Cu and Mn in liver tissue between the groups evaluated.

Spot ID	High Cu/Mn SO_4_** x** Low Cu/Mn SO_4_	Low Cu/Mn (OH)Cl** x** Low Cu/Mn SO_4_	High Cu/Mn (OH)Cl** x** High Cu/Mn SO_4_	High Cu/Mn (OH)Cl** x** Low Cu/Mn (OH)Cl
01	− 2.42665		+ 2.90812	
02	+ 2.32529		− 1.9209	
03	+ 1.91333	− 4.89508	− 2.7908	
04	+ 1.37899	+ 1.87544		− 1.85125
05	+ 1.07939			
06	+ 2.62169		− 2.71604	
07	+ 1.31895	− 1.57209		
08	+ 1.50983	− 1.71385	− 2.26661	+ 1.32697
09	+ 1.99035		− 2.19431	
10	− 1.58689		+ 1.64955	
11	+ 2.83932		− 2.3269	
12	+ 2.46884	+ 1.30077	− 1.63919	
13	+ 1.8366		− 2.15529	
14	Absent		Present	
15	Absent	+ 3.71871	Present	− 2.89582
16	+ 1.94859		− 2.77459	
17		+ 2.17561		− 1.8057
18	+ 1.50469			+ 1.9578
19			+ 1.43375	+ 1.33486
20		+ 2.09367	− 1.43008	− 1.63192
21			− 3.07186	− 2.24224
22		− 1.62981		
23		− 2.60766		+ 4.34762
24		+ 1.81972		
25		− 3.29601		+ 2.75689
26		+ 1.32911		− 1.96446
27		− 1.96106		+ 2.02609
28		+ 1.2849		
29		Absent		Present
30		Absent		Present
31				+ 1.38711
32			− 1.79011	+ 1.27324
33	Absent		Present	+ 3.00156
34				+ 1.9165

Low Cu/Mn SO_4_: 15 ppm Cu sulfate and 80 ppm Mn sulfate; High Cu/Mn SO_4_: 150 ppm Cu sulfate and 120 ppm Mn sulfate; Low Cu/Mn (OH)Cl: 15 ppm Cu hydroxychloride and 80 ppm Mn hydroxychloride; and High Cu/Mn (OH)Cl: 150 ppm Cu hydroxychloride and 120 ppm Mn hydroxychloride.

Positive and negative values indicate the abundance of protein spots up or down in the first group compared to the second. The expression present or absent refers to the presence or absence of protein spots in the first group compared to the second.

### Protein identification, functional analysis, protein‒protein interaction, and metabolic pathways

A total of 56 nonredundant proteins with a score ≥ 60 were identified from the 34 protein spots associated with Cu and Mn. The identified proteins have a molecular mass ranging from approximately 4–91 kDa, within the pH range of 3–10 (Table 4). The results of the functional annotation (biological processes, molecular functions, and cellular components) of the proteins can be seen in Supplementary Figure S2 [see Supplementary Information]. Briefly, the proteins characterized in the present study are distributed in several cellular components, such as intracellular anatomical structure, cytoplasm, cytosol, organelles, membrane, hemoglobin complex, and catalytic complex. In addition, they participate in several metabolic processes, development, biological responses, and regulation. Among the molecular functions performed by the identified proteins are binding, molecular chaperones, and transferase and hydrolase activity.

**Table 4:** Proteins identified by LC‒MS/MS in protein spots associated with Cu and Mn in liver tissue. The score values indicate the reliability of protein identification. High score values suggest greater confidence in protein identification, while very low scores indicate a less reliable match. In the study, proteins with a score > 60 were considered.

Spot	Accession	Gene	Protein	Experimental pI/MM (Da)	Theoretical pI/MM (Da)	Score	Presence of Cu and/or Mn
01	P83145	N/A	Ribonuclease UK114	8.94/15,241	5.85/4083.76	6335.108	High Cu/Mn (OH)Cl = Cu
	P01994	HBAA	Hemoglobin subunit alpha-A		8.54/15,428.87	5828.38	
	P80226	FABP1	Fatty acid-binding protein. liver		8.67/14,210.37	1769.519	
	P02112	HBB	Hemoglobin subunit beta		8.84/16,466.13	820.2869	
	P02127	N/A	Hemoglobin subunit rho		8.78/16,589.21	281.6852	
	P02128	HBE	Hemoglobin subunit épsilon		9.07/16,603.23	281.6852	
	P00337	LDHB	L-lactate dehydrogenase B chain		7.07/36,318.09	263.6445	
	P02001	HBAD	Hemoglobin subunit alpha-D		7.02/15,694.93	216.1184	
	P02007	N/A	Hemoglobin subunit pi		7.81/15,709.04	79.9931	
	Q5ZI49	PRPS2	Ribose-phosphate pyrophosphokinase 2		6.37/35,663.21	79.7743	
	P23991	ADH1	Alcohol dehydrogenase 1		8.69/39,807.50	60.1557	
02	P51913	ENO1	Alpha-enolase	5.52/27,880	6.17/47,305.01	473.2483	High Cu/Mn SO_4_ = Cu
	P17153	ANXA5	Annexin A5		5.59/36,198.34	446.3777	
	P07322	ENO3	Beta-enolase		7.28/47,196.08	360.0507	
	P14315	CAPZB	F-actin-capping protein subunit beta isoforms 1 and 2		5.36/31,364.45	221.4613	High Cu/Mn (OH)Cl = Cu
	P19121	ALB	Albumin		5.51/69,918.19	108.1769	
	P07341	ALDOB	Fructose-bisphosphate aldolase B		8.81/39,295.78	71.3977	
03	P17153	ANXA5	Annexin A5	5.87/31,101	5.59/36,198.34	5548.77	High Cu/Mn SO_4_ = Cu
	P51913	ENO1	Alpha-enolase		6.17/47,305.01	610.6372	High Cu/Mn (OH)Cl = Cu
	P07322	ENO3	Beta-enolase		7.28/47,196.08	490.8612	
04	Q9I923	RGN	Regucalcin	5.83/29,922	5.77/33,229.57	8090.358	High Cu/Mn SO_4_ = Mn
	P51913	ENO1	Alpha-enolase		6.17/47,305.01	239.0043	High Cu/Mn (OH)Cl = Mn
	P21872	GART	Trifunctional purine biosynthetic protein adenosine-3		7.51/106,544	119.1895	
05	P00337	LDHB	L-lactate dehydrogenase B chain		7.07/36,318.09	8487.15	High Cu/Mn SO_4_ = Cu
	P07341	ALDOB	Fructose-bisphosphate aldolase B		8.81/39,295.78	4702.678	High Cu/Mn (OH)Cl = Cu
	P23991	ADH1	Alcohol dehydrogenase 1		8.69/39,807.5	196.0894	
06	Q5ZMQ2	ACTG1	Actin, cytoplasmic 2	5.47/37,266	5.31/41,792.84	8439.855	High Cu/Mn SO_4_ = Cu
	P53478	N/A	Actin, cytoplasmic type 5		5.30/41,835.87	8439.855	
	P60706	ACTB	Actin, cytoplasmic 1		5.29/41,736.73	8439.855	
	P68139	ACTA1	Actin, alpha skeletal muscle		5.23/42,051.03	3354.721	
	P68034	ACTC1	Actin, alpha cardiac muscle 1		5.23/42,018.97	3354.721	High Cu/Mn (OH)Cl = Cu
	P63270	ACTG2	Actin, gamma-enteric smooth muscle		5.31/41,876.88	3354.721	
	P08023	ACTA2	Actin, aortic smooth muscle		5.24/41,994.92	3354.721	
	P19121	ALB	Albumin		5.51/69,918.19	83.924	
07	P00356	GAPDH	Glyceraldehyde-3-phosphate dehydrogenase	7.86/31,789	8.70/35,703.97	8609.476	High Cu/Mn SO_4_ = Cu
	P07341	ALDOB	Fructose-bisphosphate aldolase B		8.81/39,295.78	68.7995	High Cu/Mn (OH)Cl = Cu
08	P16580	GLUL	Glutamine synthetase	7.09/46,191	6.38/42,146.46	1430.712	High Cu/Mn SO_4_ = Mn
							High Cu/Mn (OH)Cl = Mn
09	P51913	ENO1	Alpha-enolase	6.52/46,594	6.17/47,305.01	380.7553	High Cu/Mn SO_4_ = Mn
	P07322	ENO3	Beta-enolase		7.28/47,196.08	247.165	High Cu/Mn (OH)Cl = Mn
	P27463	ALDH1A1	Retinal dehydrogenase 1		7.49/55,809.33	66.4081	
10	O93344	ALDH1A2	Retinal dehydrogenase 2		5.87/56,731.99	66.4081	
	P51913	ENO1	Alpha-enolase	6.65/43,874	6.17/47,305.01	10,775.61	High Cu/Mn SO_4_ = CuHigh Cu/Mn (OH)Cl = Cu
	O57391	ENO2	Gamma-enolase		4.84/47,308.46	2788.5	
	P07322	ENO3	Beta-enolase		7.28/47,196.08	1849.809	
11	P51913	ENO1	Alpha-enolase	4.26/58,283	6.17/47,305.01	559.8503	High Cu/Mn (OH)Cl = Cu
	P07322	ENO3	Beta-enolase		7.28/47,196.08	555.7766	
12	P09102	P4HB	Protein disulfide-isomerase	4.65/60,297	4.69/57,409.80	5805.812	High Cu/Mn SO_4_ = Cu
	P08110	HSP90B1	Endoplasmin		4.83/91,555.02	1379.731	
							High Cu/Mn (OH)Cl = Cu
	Q04619	HSP90AB1	Heat shock cognate protein HSP 90-beta		4.94/83,427.45	142.0041	High Cu/Mn SO_4_ = Cu
13	Q5ZL72	HSPD1	60 kDa heat shock protein, mitochondrial	5.46/62,486	5.72/60,972.56	13,474.97	
	Q5ZM98	HSPA9	Stress-70 protein, mitochondrial		6.09/73,192.32	122.2014	High Cu/Mn (OH)Cl = Cu
14	P51913	ENO1	Alpha-enolase	6.00/45,904	6.17/47,305.01	3681.357	High Cu/Mn (OH)Cl = Cu e Mn
	P07322	ENO3	Beta-enolase		7.28/47,196.08	523.2764	
	O57391	ENO2	Gamma-enolase		4.84/47,308.46	299.1514	
15	P51913	ENO1	Alpha-enolase	6.31/45,224	6.17/47,305.01	4588.928	High Cu/Mn (OH)Cl = Cu e Mn
	O57391	ENO2	Gamma-enolase		4.84/47,308.46	978.15	
	P07322	ENO3	Beta-enolase		7.28/47,196.08	771.7178	
16	P19121	ALB	Albumin	5.4/74,696	5.51/69,918.19	3543.291	High Cu/Mn (OH)Cl = Mn
	Q90593	HSPA5	Endoplasmic reticulum chaperone BiP		5.12/72,018.51	2434.631	
	O73885	HSPA8	Heat shock cognate 71 kDa protein		5.46/70,826.98	236.3526	
	P08106	N/A	Heat shock 70 kDa protein		5.52/69,750.82	214.6749	
17	P02001	HBAD	Hemoglobin subunit alpha-D	6.52/14,400	7.02/15,694.93	4248.779	High Cu/Mn (OH)Cl = Cu
	P00356	GAPDH	Glyceraldehyde-3-phosphate dehydrogenase		8.70/35,703.97	2980.18	
	P80566	SOD1	Superoxide dismutase [Cu–Zn]		6.1/15,703.6	1844.127	
	P02112	HBB	Hemoglobin subunit beta		8.84/16,466.13	890.2412	
	P07341	ALDOB	Fructose-bisphosphate aldolase B		8.81/39,295.78	570.7814	
	P02128	HBE	Hemoglobin subunit epsilon		9.07/16,603.23	208.8738	
	P02127	N/A	Hemoglobin subunit rho		8.78/16,589.21	175.0719	
	P80226	FABP1	Fatty acid-binding protein, liver		8.67/14,210.37	127.1781	
18	P20136	GSTM2	Glutathione S-transferase 2	7.73/26,038	6.84/25,892.82	8173.118	High Cu/Mn SO_4_ = Cu
	P07341	ALDOB	Fructose-bisphosphate aldolase B		8.81/39,295.78	1314.413	
	P02001	HBAD	Hemoglobin subunit alpha-D		7.02/15,694.93	1045.607	
	P00356	GAPDH	Glyceraldehyde-3-phosphate dehydrogenase		8.70/35,703.97	625.0613	High Cu/Mn (OH)Cl = Cu
	Q5ZME2	MDH1	Malate dehydrogenase, cytoplasmic		6.92/36,543.40	283.7447	
	P47826	RPLP0	60S acidic ribosomal protein P0		5.71/34,285.58	175.4232	
19	P00356	GAPDH	Glyceraldehyde-3-phosphate dehydrogenase	8.28/29,455	8.70/35,703.97	12,218.79	High Cu/Mn SO_4_ = Mn
	P07341	ALDOB	Fructose-bisphosphate aldolase B		8.81/39,295.78	186.3181	
							High Cu/Mn (OH)Cl = Cu e Mn
	P23991	ADH1	Alcohol dehydrogenase 1		8.69/39,807.50	168.1874	
20	Q5ZMQ2	ACTG1	Actin, cytoplasmic 2	5.35/41,857	5.31/41,792.84	16,243.58	High Cu/Mn SO_4_ = Cu
	P60706	ACTB	Actin, cytoplasmic 1		5.29/41,736.73	16,243.58	
	P53478	N/A	Actin, cytoplasmic type 5		5.30/41,835.87	16,238.88	
	P68034	ACTC1	Actin, alpha cardiac muscle 1		5.23/42,018.97	5004.212	
	P68139	ACTA1	Actin, alpha skeletal muscle		5.23/42,051.03	4902.417	High Cu/Mn (OH)Cl = Cu
	P63270	ACTG2	Actin, gamma-enteric smooth muscle		5.31/41,876.88	4859.32	
	P08023	ACTA2	Actin, aortic smooth muscle		5.24/41,994.92	4852.473	
	P19121	ALB	Albumin		5.51/69,918.19	2513.817	
	P00356	GAPDH	Glyceraldehyde-3-phosphate dehydrogenase		8.70/35,703.97	202.1998	
	Q5ZL72	HSPD1	60 kDa heat shock protein, mitochondrial		5.72/ 60,972.56	198.1112	
21	P19121	ALB	Albumin	5.61/72,669	5.51/69,918.19	11,922.4	High Cu/Mn SO_4_ = Cu
	O73885	HSPA8	Heat shock cognate 71 kDa protein		5.46/70,826.98	2878.664	High Cu/Mn (OH)Cl = Cu
	P08106	N/A	Heat shock 70 kDa protein		5.52/69,750.82	997.7423	
	Q90593	HSPA5	Endoplasmic reticulum chaperone BiP		5.12/72,018.51	394.2814	
	Q5ZM98	HSPA9	Stress-70 protein, mitochondrial		6.09/73,192.32	152.2754	
22	P19121	ALB	Albumin	7.42/54,093	5.51/69,918.19	244.9902	High Cu/Mn SO_4_ = Mn
	P00368	GLUD1	Glutamate dehydrogenase 1, mitochondrial		8.48/55,712.06	163.0693	
							High Cu/Mn (OH)Cl = Mn
23	Q08392	N/A	Glutathione S-transferase	9.45/25,785	8.86/25,298.52	1478.593	High Cu/Mn (OH)Cl = Cu
	Q08393	N/A	Glutathione S-transferase		8.76/25,413.73	1474.454	
	P26697	N/A	Glutathione S-transferase 3		9.06/26,325.84	548.1452	
	P00508	GOT2	Aspartate aminotransferase, mitochondrial		9.39/47,241.27	371.8729	
	P00356	GAPDH	Glyceraldehyde-3-phosphate dehydrogenase		8.70/35,703.97	336.4139	
	Q5ZL72	HSPD1	60 kDa heat shock protein, mitochondrial		5.72/60,972.56	198.1112	
24	P07341	ALDOB	Fructose-bisphosphate aldolase B	8.11/34,234	8.81/39,295.78	6992.838	High Cu/Mn (OH)Cl = Mn
	P00356	GAPDH	Glyceraldehyde-3-phosphate dehydrogenase		8.70/35,703.97	2426.886	
	P23991	ADH1	Alcohol dehydrogenase 1		8.69/39,807.50	1040.125	
	P00337	LDHB	L-lactate dehydrogenase B chain		7.07/36,318.09	147.8423	
25	P00368	GLUD1	Glutamate dehydrogenase 1, mitochondrial	7.82/51,213	8.48/55,712.06	2828.28	High Cu/Mn SO_4_ = Cu e Mn
	P27463	ALDH1A1	Retinal dehydrogenase 1		7.49/55,809.33	2341.783	
	P07341	ALDOB	Fructose-bisphosphate aldolase B		8.81/39,295.78	299.201	High Cu/Mn (OH)Cl = Cu e Mn
	O93344	ALDH1A2	Retinal dehydrogenase 2		5.87/56,731.99	155.6904	
26	Q5ZJF4	PRDX6	Peroxiredoxin-6	6.65/25,240	5.7/24,976.79	6267.357	High Cu/Mn (OH)Cl = Mn
	P07341	ALDOB	Fructose-bisphosphate aldolase B		8.81/39,295.78	1609.665	
	P21642	PCK2	Phosphoenolpyruvate carboxykinase [GTP], mitochondrial		7.56/71,106.29	878.0714	
	P16580	GLUL	Glutamine synthetase		6.38/42,146.46	799.0565	
	P38024	AIRC	Multifunctional protein ADE2		8.18/47,240.32	279.2373	
	P19121	ALB	Albumin		5.51/69,918.19	93.6207	
	Q5ZLR5	UQCRFS1	Cytochrome b-c1 complex subunit Rieske, mitochondrial		8.68/29,386.56	92.6274	
	P00940	TPI1	Triosephosphate isomerase		6.71/26,620.45	91.4796	
27	P07341	ALDOB	Fructose-bisphosphate aldolase B	8.59/39,065	8.81/39,295.78	98.4781	High Cu/Mn (OH)Cl = Cu
28	P07341	ALDOB	Fructose-bisphosphate aldolase B	8.6/35,327	8.81/39,295.78	17,119.69	High Cu/Mn SO_4_ = Cu
	P23991	ADH1	Alcohol dehydrogenase 1		8.69/39,807.50	1166.58	
	P00356	GAPDH	Glyceraldehyde-3-phosphate dehydrogenase		8.70/35,703.97	838.7416	
	Q5ZKR4	RABL3	Rab-like protein 3		6.23/26,002.35	94.9377	
	P00340	LDHA	L-lactate dehydrogenase A chain		7.75/36,514.46	73.7079	
	P00337	LDHB	L-lactate dehydrogenase B chain		7.07/36,318.09	64.6321	
29	P07341	ALDOB	Fructose-bisphosphate aldolase B	9.31/21,742	8.81/39,295.78	1455.896	High Cu/Mn (OH)Cl = Cu
	Q08392	N/A	Glutathione S-transferase		8.86/25,298.52	867.9658	
	P26697	N/A	Glutathione S-transferase 3		9.06/26,325.84	664.4908	
	Q08393	N/A	Glutathione S-transferase		8.76/25,413.73	308.8971	
30	P00356	GAPDH	Glyceraldehyde-3-phosphate dehydrogenase	7.95/36,340	8.70/35,703.97	5820.609	High Cu/Mn (OH)Cl = Mn
	P07341	ALDOB	Fructose-bisphosphate aldolase B		8.81/39,295.78	522.2266	
	P00337	LDHB	L-lactate dehydrogenase B chain		7.07/36,318.09	326.4798	
	P00940	TPI1	Triosephosphate isomerase		6.71/26,620.45	250.532	
31	P51913	ENO1	Alpha-enolase	4.25/76,181	6.17/47,305.01	642.0277	High Cu/Mn (OH)Cl = Mn
	P07322	ENO3	Beta-enolase		7.28/47,196.08	545.8586	
	O57391	ENO2	Gamma-enolase		4.84/47,308.46	91.4921	
32	P60706	ACTB	Actin, cytoplasmic 1	5.25/41,900	5.29/41,736.73	16,763.91	High Cu/Mn SO_4_ = Cu
	Q5ZMQ2	ACTG1	Actin, cytoplasmic 2		5.31/41,792.84	16,745.55	
	P53478	N/A	Actin, cytoplasmic type 5		5.30/41,835.87	16,742.94	
	P68139	ACTA1	Actin, alpha skeletal muscle		5.23/42,051.03	4365.88	High Cu/Mn (OH)Cl = Cu
	P68034	ACTC1	Actin, alpha cardiac muscle 1		5.23/42,018.97	4365.88	
	P08023	ACTA2	Actin, aortic smooth muscle		5.24/41,994.92	4193.435	
	P63270	ACTG2	Actin, gamma-enteric smooth muscle		5.31/41,876.88	4170.822	
	P19121	ALB	Albumin		5.51/69,918.19	600.1381	
33	P00940	TPI1	Triosephosphate isomerase	7.74/26,230	6.71/26,620.45	15,437.87	High Cu/Mn (OH)Cl = Mn
	P07341	ALDOB	Fructose-bisphosphate aldolase B		8.81/39,295.78	1635.883	
	P31335	ATIC	Bifunctional purine biosynthesis protein ATIC		8.40/64,414.82	175.2742	
34	Q90593	HSPA5	Endoplasmic reticulum chaperone BiP	5.22/69,732	5.12/72,018.51	9989.707	High Cu/Mn (OH)Cl = Cu e Mn
	P08106	N/A	Heat shock 70 kDa protein		5.52/69,750.82	853.5319	
	O73885	HSPA8	Heat shock cognate 71 kDa protein		5.46/70,826.98	849.6843	
	P07341	ALDOB	Fructose-bisphosphate aldolase B		8.81/39,295.78	166.5883	

MM: Molecular Mass; High Cu/Mn SO_4_: 150 ppm Cu sulfate and 120 ppm Mn sulfate; and High Cu/Mn (OH)Cl: 150 ppm Cu hydroxychloride and 120 ppm Mn hydroxychloride.

**Figure 2:**
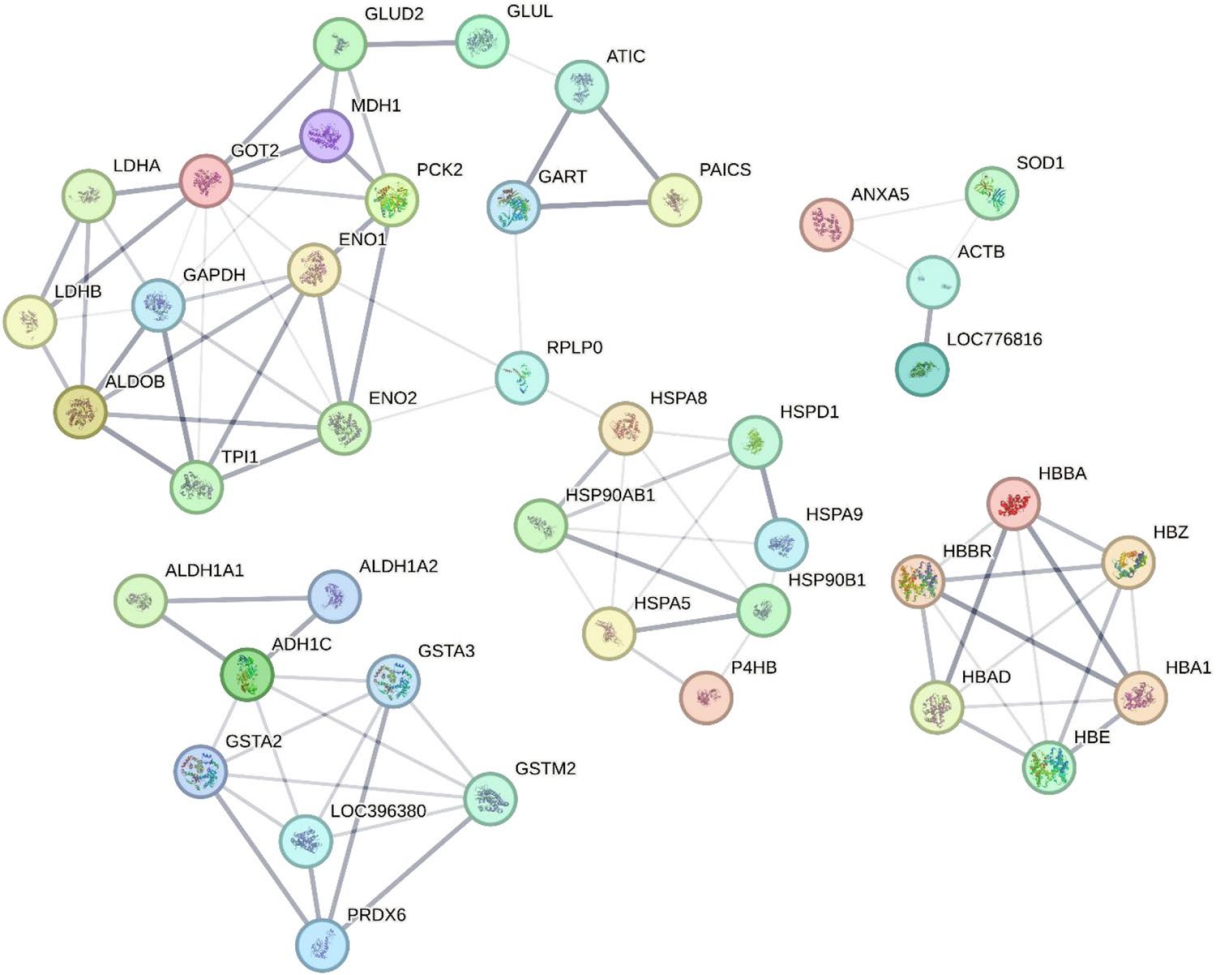
Protein‒protein interaction network of proteins identified in protein spots associated with copper and manganese. The network was generated using the String online database. Each node (colored spheres) represents a protein. The lines represent evidence of interaction between the connected proteins, and the thickness of the lines indicates the confidence level of the respective interactions (the thicker the line is, the greater the confidence level). ACTB (Actin, cytoplasmic 1), ADH1C (Alcohol dehydrogenase (1), ALB (Serum albumin), ALDH1A1 (Retinal dehydrogenase 1), ALDH1A2 (Retinal dehydrogenase (2), ALDOB (Fructose-bisphosphate aldolase B), ANXA5 (Annexin A5), ATIC (Bifunctional purine biosynthetic protein ATIC), ENO1 (Alpha-enolase), ENO2 (Gamma-enolase), GAPDH (Glyceraldehyde-3-phosphate dehydrogenase), GART (Trifunctional purine biosynthetic protein adenosine-3), GLUD2 (Glutamate dehydrogenase 1, mitochondrial), GLUL (Glutamine synthetase), GOT2 (Aspartate aminotransferase, mitochondrial), GSTA2 (Glutathione S-transferase), GSTA3 (Glutathione S-transferase), GSTM2 (Glutathione S-transferase 2), HBA1 (Hemoglobin subunit alpha-A), HBAD (Hemoglobin subunit alpha-D), HBBA (Hemoglobin subunit beta), HBBR (Hemoglobin subunit rho), HBE (Hemoglobin subunit epsilon), HBZ (Hemoglobin subunit pi), HSP90AB1 (Heat shock cognate protein HSP 90-beta), HSP90B1 (Endoplasmin), HSPA5 (Endoplasmic reticulum chaperone BiP), HSPA8 (Heat shock cognate 71 kDa protein), HSPA9 (Stress-70 protein, mitochondrial), HSPD1 (60 kDa heat shock protein, mitochondrial), LBFABP (Fatty acid-binding protein, liver), LDHA (L-lactate dehydrogenase A chain), LDHB (L-lactate dehydrogenase B chain), LOC396380 (Glutathione S-transferase 3), LOC776816 (Actin, cytoplasmic 2), MDH1 (Protein disulfide-isomerase), P4HB (Protein disulfide-isomerase), PAICS (Multifunctional protein ADE2), PCK2 (Phosphoenolpyruvate carboxykinase [GTP], mitochondrial), PRDX6 (Peroxiredoxin-6), PRPS2 (Ribose-phosphate pyrophosphokinase 2), RGN (Regucalcin), RPLP0 (60S acidic ribosomal protein P0), SOD1 (Superoxide dismutase [Cu–Zn]), and TPI1 (Triosephosphate isomerase).

The protein-interaction network generated via String is illustrated in Fig. 2. The network shows the interaction between 45 proteins, where each protein is represented by a node (or circle) colored with the name of the gene associated with it. The lines indicate evidence of interaction between proteins that act together for related functions. In the image it is possible to observe the interaction of molecular chaperones responsible for the folding and refolding of proteins (HSP90AB1, HSP90B1, HSPA5, HSPA8, HSPA9 and HSPD1). Interactions are also observed between proteins involved in various metabolic processes, such as carbohydrate metabolism (GAPDH, ENO1, ENO2, PCK2, ALDOB, TPI1, LDHB and LDHA), metabolism of purines (ATIC, GART and PAICS), xenobiotic metabolism (ADH1C, GSTA2, GSTA3, GSTM2 and LOC396380) and oxygen transport (HBA1, HBAD, HBBA, HBBR, HBE and HBZ).

A total of 29 KEGG pathways were significantly enriched (FDR < 0.05) using Cytoscape software with the string plugin (Table 5). The 10 most enriched pathways were Metabolic pathways (gga01100), Glycolysis/Gluconeogenesis (gga00010), Carbon metabolism (gga01200), Biosynthesis of amino acids (gga01230), Drug metabolism—cytochrome P450 (gga00982), Metabolism of xenobiotics by cytochrome P450 (gga00980), Pyruvate metabolism (gga00620), Protein processing in endoplasmic reticulum (gga04141), Cysteine and methionine metabolism (gga00270) and Glutathione metabolism (gga00480).

**Table 5:** KEGG pathways significantly enriched (FDR < 0.05) using Cytoscape software with the String plugin.

Pathway	Description	FDR*	Genes
gga01100	Metabolic pathways	1.87E-13	GLUD1|GOT2**|**ENO1|FASN|PRDX6|ATIC|GLUL|MDH1|PCK2|ADH1C|PAICS|GAPDH|TPI1|ENO2|ALDH1A1|LOC396380|ENSGALP00000026286|GSTA|PRPS2|RGN|ALDH1A2|GART|ALDOB |LDHA|LDHB|UQCRFS1
gga00010	Glycolysis/Gluconeogenesis	1.99E-11	ENO1|PCK2|ADH1C|GAPDH|TPI1|ENO2|ALDOB|LDHA|LDHB
gga01200	Carbon metabolism	1.43E-10	GLUD1|GOT2|ENO1|MDH1|GAPDH|TPI1|ENO2|PRPS2|RGN|ALDOB
gga01230	Biosynthesis of amino acids	1.88E-09	GOT2|ENO1|GLUL|GAPDH|TPI1|ENO2|PRPS2|ALDOB
gga00982	Drug metabolism—cytochrome P450	1.00E-04	ADH1C|LOC396380|ENSGALP00000026286|GSTA
gga00980	Metabolism of xenobiotics by cytochrome P450	1.30E-04	ADH1C|LOC396380|ENSGALP00000026286|GSTA
gga00620	Pyruvate metabolism	1.40E-04	MDH1|PCK2|LDHA|LDHB
gga04141	Protein processing in endoplasmic reticulum	1.70E-04	HSPA5|HSPA8|P4HB|HSP90AB1|HSPA2|HSP90B1
gga00270	Cysteine and methionine metabolism	2.50E-04	GOT2|MDH1|LDHA|LDHB
gga00480	Glutathione metabolism	2.50E-04	PRDX6|LOC396380|ENSGALP00000026286|GSTA
gga00220	Arginine biosynthesis	5.60E-04	GLUD1|GOT2|GLUL
gga03018	RNA degradation	0.0011	HSPA9|ENO1|HSPD1|ENO2
gga00030	Pentose phosphate pathway	0.0012	PRPS2|RGN|ALDOB
gga00830	Retinol metabolism	0.0023	ADH1C|ALDH1A1|ALDH1A2
gga00250	Alanine, aspartate and glutamate metabolism	0.0026	GLUD1|GOT2|GLUL
gga00230	Purine metabolism	0.0061	ATIC|PAICS|PRPS2|GART
gga00983	Drug metabolism—other enzymes	0.0064	LOC396380|ENSGALP00000026286|GSTA
gga04520	Adherens junction	0.0093	ACTB|ACTG1|LOC776816
gga00670	One carbon pool by folate	0.0156	ATIC|GART
gga00910	Nitrogen metabolism	0.0156	GLUD1|GLUL
gga00020	Citrate cycle (TCA cycle)	0.0307	MDH1|PCK2
gga00350	Tyrosine metabolism	0.0359	GOT2|ADH1C
gga00051	Fructose and mannose metabolism	0.0366	TPI1|ALDOB
gga00630	Glyoxylate and dicarboxylate metabolism	0.0366	GLUL|MDH1
gga00640	Propanoate metabolism	0.0366	LDHA|LDHB
gga04210	Apoptosis	0.0366	ACTB|ACTG1|LOC776816
gga04217	Necroptosis	0.0366	GLUD1|GLUL|HSP90AB1
gga04145	Phagosome	0.0479	ACTB|ACTG1|LOC776816
gga04530	Tight junction	0.0484	ACTB|ACTG1|LOC776816

*FDR: False Discovery Rate.

## Discussion

### Cu and Mn concentrations in liver tissue and protein pellet

The concentrations of Cu and Mn in the liver tissue and protein pellet of the high Cu/Mn SO_4_ and high Cu/Mn (OH)Cl groups were higher than those of the low Cu/Mn SO_4_ and low Cu/Mn (OH)Cl groups, probably due to the greater supplementation of these minerals. Kim and Kil^10^ also observed an increase in Cu concentrations in the liver of broiler chickens with increasing inclusion of CuSO_4_ and tribasic copper chloride (TBCC) in the diets. Similarly, Sun et al^11^. reported higher Mn concentrations in various broiler tissues as the supplemental Mn hydroxychloride level was increased.

Comparing the supplementation sources, it was observed that Cu hydroxychloride proved to be more bioavailable than Cu sulfate, since its use resulted in higher concentrations of Cu in the hepatic tissue at the level of 15 ppm and in the protein pellet at both levels (15 and 150 ppm). Although the Mn concentrations did not differ between the sources in the liver tissue samples, in the protein pellet, there was a higher concentration of Mn in the group supplemented with Mn hydroxychloride at a level of 80 ppm compared to the sulfate source with the same level. In this sense, the analysis of mineral concentration in the protein pellet proved to be more sensitive to estimate bioavailability in relation to the concentration in tissue samples. These results suggest that in the present study, there was a greater incorporation of Cu and Mn in the liver proteins of broiler chickens supplemented with hydroxychlorides. In fact, the analysis by GFAAS indicated a greater number of protein spots with the presence of Cu and Mn in the gels of the high Cu/Mn (OH)Cl group compared to the high Cu/Mn SO_4_ group, which reinforces the hypothesis that hydroxychlorides may be more efficient in making absorbed minerals available for use by proteins in relation to sulfates.

### Cu and Mn mapping in protein spots

Analysis by GFAAS indicated the unique presence of Cu in 19 spots. When analyzing the proteins expressed in these spots, it was observed that 5 spots showed the expression of the copper-binding protein albumin (spots 02, 06, 20, 21 and 32), and 1 spot showed the expression of the metalloenzyme of Cu and Zn, superoxide dismutase [Cu–Zn] (spot 17). Other proteins known to bind to copper, such as ATOX1 copper chaperones and CCs, were not identified, possibly due to the limitations presented by the 2D-PAGE technique in resolving low-abundance proteins and the dye used to reveal the protein spots. According to Smith et al^12^., low abundance proteins are hardly detected by Coomassie Blue staining, whose limit is between 50 and 100 ng of protein.

On the other hand, in 13 spots with the sole presence of Cu and 5 spots with the concomitant presence of Cu and Mn, the expression of proteins was observed that, as reported in the literature thus far, do not depend exclusively on Cu to perform their functions. However, some expressed proteins are metalloproteins activated by divalent metals, such as enolases (ENO1, ENO2 and ENO3) and alcohol dehydrogenase 1 (ADH1). Enolases are metalloenzymes that participate in the glycolytic pathway and are preferentially activated by Mg^2+^. ADH1 is part of a class of Zn^2+^-dependent enzymes responsible for the oxidation and reduction of a wide variety of alcohols and aldehydes^13^. These enzymes are extremely abundant in the liver, and in the present study, they were identified in several protein spots that showed divergent abundance. For this reason, it was not possible to evaluate the difference in the expression of these proteins between the groups studied using the 2D-PAGE technique. However, the presence of Cu in the spots where enolases (spots 02, 03, 10, 11, 14 and 15) and ADH1 (spots 01, 05, 28 and 19) were identified may indicate a possible replacement of Mg^2+^ and Zn^2+^ by Cu^2+^ when supplemented at levels higher than recommended.

Curiously, in 7 spots (spots 07, 12, 13, 18, 23, 27 and 29) with the sole presence of Cu and 2 spots (spots 25 and 34) with the simultaneous presence of Cu and Mn, proteins that normally do not are known to exhibit metal binding. These include proteins involved in carbohydrate metabolism (GAPDH, ALDOB, MDH1), retinol metabolism (ALDH1A1, ALDH1A2), protein folding and refolding (HSP90B1, HAP90AB1, HSPD1, HSPA9, HSPA5, HSPA8, Heat shock 70 kDa protein), detoxification (P4HB, GSTM2, Glutathione-s-transferase), and other processes (GLUD1, GOT2, RPLP0). This may have occurred due to the affinity of Cu^+^ (reduced state) for thiol and thioether groups found in cysteine and methionine residues and of Cu^2+^ (oxidized state) for oxygen groups found in aspartic and glutamic acid or imidazole nitrogen in residues of histidine^14^. This property enables the interaction of copper with a wide range of proteins, establishing their functions and structural states, in addition to causing harmful effects in cases of excess metal^15^.

Smith et al^12^. performed a study using metal affinity chromatography (IMAC), 2D-PAGE and mass spectrometry to identify human hepatocellular proteins with copper binding capacity. The authors identified 19 microsomal proteins and 48 cytosolic proteins with copper binding capacity. Among them are some proteins homologous to those reported in this study, such as protein disulfide isomerase, glyceraldehyde-3-phosphate dehydrogenase, heat shock protein 60 kD, heat shock cognate 71 kD protein, endoplasmic reticulum chaperone BiP and aspartate aminotransferase*.* In addition to these proteins, proteins commonly known to bind to metals were detected, such as albumin (Cu), alcohol dehydrogenase (Zn), α-enolase (Mg) and annexin V (Ca), which were also identified in our study. According to the authors, it is unlikely that all identified proteins bind to copper under normal physiological conditions; however, some proteins may be targets for copper under conditions of elevated levels.

Manganese was identified in only 10 spots and in 5 spots simultaneously with Cu. In 3 spots (spots 04, 09 and 31) with the sole presence of Mn and 2 spots (14 and 15) with Cu and Mn together, the previously mentioned enolases were characterized, indicating that, similar to Cu^2+^, Mn^2+^ can replace Mg^2+^ in these proteins. Other studies have shown that Mn^2+^ can replace Mg^2+^ in active sites of many proteins^16–20^. This substitution can occur because Mn^2+^ is a hard Lewis acid, similar to Mg^2+^, which allows the inverse to also occur^21^. In spots 19 and 24, ADH1 was characterized, indicating that it is a protein with the potential to bind Mn, although Cu has shown more affinity due to the greater number of spots associated with this metal and the respective protein.

Regarding the known Mn-binding proteins, 5 proteins were identified, namely, regucalcin (spot 04), trifunctional purine biosynthetic protein adenosine-3 (spot 04), glutamine synthetase (spot 08 e 26), phosphoenolpyruvate carboxykinase [GTP], and mitochondrial (spot 26). Albumin was identified in 3 spots with the unique presence of Mn (spots 16, 22 and 26). Although albumin is best known for binding to physiological Cu^2+^ and Zn^2+^ and to toxic Ni^2+^ and Cd^2+^^22^, there are reports in the literature that Mn^2+^ has two binding sites in albumin, where the secondary binding of Mn^2+^ corresponds to the primary binding site of Zn^2+^^23^, which justifies the identification of albumin in spots with Mn.

Similar to the spots with Cu, in the spots associated solely with Mn, proteins that have no known direct relationship with the metal were also characterized, such as HSPA5, HSPA8, Heat shock 70 kDa protein, ALDH1A1, ALDH1A2, GAPDH, ALDOB, GLUD1, LDHB, TPI1 and ATIC (spots 09, 16, 22, 24, 30 and 33). Normally, Mn^2+^ forms relatively weak complexes with many ligands compared to Cu^2+^^21^, which was reflected in the smaller number of spots associated with Mn. However, the concentration of 120 ppm Mn in the diet may have favored its binding to different proteins and consequently its detection in protein spots.

### Changes in protein regulation in response to different sources and elevated levels of Cu and Mn

In the vast majority of protein spots, more than one protein was characterized, and many proteins were identified in more than one spot. According to Zhang et al^24^., a protein can be identified in several spots distributed in different positions on the gel due to posttranslational modifications that cause changes in pI and molecular mass. This fact explains the identification of the same protein in multiple spots in the present study and suggests that these posttranslational modifications may be involved in the response to Cu and Mn supplementation above nutritional levels. However, this factor made it difficult to assess the regulation of most of the proteins (upregulated or downregulated) between the studied groups, since divergent abundance of the respective spots were found. For example, analysis of the KEGG pathways revealed that one of the altered pathways was the glycolysis and gluconeogenesis pathways. However, the wide distribution of the proteins involved in these pathways in the protein spots does not allow any inference about their relationship with the evaluated treatments. Thus, the discussion was based on proteins identified in the spots that showed consistent abundance.

From the results of the present study, it is not possible to assume that the levels of 150 ppm Cu and 120 ppm Mn caused toxic effects in the broilers. However, Cu and Mn supplementation above the nutritional recommendation altered the abundance of spots containing proteins involved in many metabolic pathways, indicating that a homeostatic imbalance may have occurred that triggered the activation of several mechanisms for restoring homeostasis.

The heat shock proteins (HSP90B1, HSP90AB1, HSPD1, HSPA9, HSPA5, HSPA8, Heat shock 70 kDa protein) known as HSPs were upregulated (spots 12, 13 and 16) in the group supplemented with Cu and Mn sulfate at levels higher (high Cu/Mn SO_4_) than the group supplemented according to dietary requirements (low Cu/Mn SO_4_). HSPs are involved in the stress response and are sensitive to various stressors, such as oxidants, toxins, toxic metals, and free radicals. These molecular chaperones play a key role in the correct folding of proteins, refolding of misfolded proteins, prevention of cytotoxic aggregates or elimination of damaged proteins during cellular stress^25^. In this sense, HSPs may have been induced to suppress the effects of possible oxidative stress generated by Cu and Mn supplementation above the nutritional recommendation, since high levels can trigger the formation of reactive oxygen species (e.g., Fenton's reaction and Haber–Weiss) and alter the normal functioning of many proteins for which they have binding sites.

Glutathione-s-transferases (GSTs) are enzymes that have multiple functions and play a key role in protecting against oxidative stress, acting mainly in the detoxification of various compounds in the liver, such as xenobiotics, lipid peroxidation products and metal ions^26–29^. In the group supplemented with high levels of Cu and Mn from the hydroxychloride source, an upregulation of GST and its isoforms was observed in relation to the group supplemented with normal levels (spots 18 and 23). These results suggest that increased exposure to Cu and Mn induced the expression of GSTs, which in turn activated cytochrome P450 drug and xenobiotic metabolism pathways to control the harmful effects of mineral supplementation above requirements. Thus, considering the results of the present study, GSTs and HSPs seem to be involved in tolerance to Cu and Mn stress, proving to be potential candidates for biomarkers of high exposure of these metals in broiler chickens. Further studies are needed to investigate the sensitivity and response of these proteins to metals individually, as well as to different sources of mineral supplementation.

The results of the mapping of Cu and Mn in the protein spots of the high Cu/Mn SO_4_ and high Cu/Mn (OH)Cl groups demonstrated that the hydroxychlorides allowed the greater incorporation of these minerals in the proteins in relation to the sulfates. On the other hand, a lower abundance of most protein spots in the high Cu/Mn (OH)Cl group was observed when compared to the high Cu/Mn SO_4_ group. Considering that the levels of Cu and Mn were above the requirements in both groups and that the hydroxychlorides proved to be more bioavailable than the sulfates, it is likely that the greater availability of minerals by the hydroxychlorides caused changes in the proteins in which Cu and Mn bound and consequently altered the functioning of these proteins, resulting in lower expression when compared to sulfates. Thus, the effects of high levels of metals from more bioavailable sources, such as hydroxychlorides, become prominent in relation to less bioavailable sources.

### Conclusions

In the present study, Cu and Mn hydroxychlorides showed greater bioavailability compared to sulphates, as indicated by the concentrations of these minerals in liver tissue samples, pellets and protein spots. In addition, supplementation above nutritional requirements induced the expression of heat shock proteins (HSPs) and detoxification proteins (GSTs), suggesting the involvement of these proteins in metal tolerance and stress. However, it is worth noting that these findings were obtained under controlled laboratory conditions. Therefore, further studies are needed taking into account the practical conditions of poultry production, since the bioavailability of minerals and protein expression can be influenced by a variety of factors, including environmental, dietary and genetic interactions. It is also recommended to study additional supplementary levels and combine different molecular techniques to obtain complementary data and a more comprehensive understanding of the results.

## Methods

The research was approved by the Ethics and Use of Animals Committee (CEUA) of the School of Veterinary Medicine and Animal Science of the São Paulo State University (UNESP), Botucatu Campus, under protocol CEUA 0191/2018. All methods were conducted in compliance with the standards issued by the National Council for Animal Experimentation Control (CONCEA) and ARRIVE (Animal Research: Reporting of In Vivo Experiments) guidelines.

### Animals, treatments, and facilities

Liver tissue samples obtained from 40 male broiler chickens (10 chickens per treatment) of the Cobb® 500 strain fed with two sources (sulfate and hydroxychloride) and two levels of copper (15 and 150 ppm) and manganese (80 and 120 ppm) were used, totaling four treatments:

- Low Cu/Mn SO_4_: 15 ppm Cu sulfate and 80 ppm Mn sulfate
- High Cu/Mn SO_4_: 150 ppm Cu sulfate and 120 mg ppm Mn sulfate
- Low Cu/Mn (OH)Cl : 15 ppm Cu hydroxychloride and 80 ppm Mn hydroxychloride
- High Cu/Mn (OH)Cl : 150 ppm Cu hydroxychloride and 120 ppm Mn hydroxychloride

The levels of 15 ppm Cu and 80 ppm Mn are in accordance with the recommendations of the Brazilian Tables for Poultry and Swine^30^, while the levels of 150 ppm Cu and 120 ppm Mn are levels above the recommendation. The diets were formulated based on corn and soybean meal and were divided into four phases, as recommended by Rostagno et al^30^.: pre-starter (1–7 d); starter (8–21 d); grower (22–35 d); and finisher (35–42 d) [see Supplementary Table S1 online]. All birds received feed and water ad libitum throughout the experimental period.

The birds were housed in 2.0 m^2^ boxes equipped with a tube feeder and a nipple drinker lined with wood shavings reused during the period from 1 to 43 days of age. The initial heating was carried out using infrared lamps (250 watts), and at the end of the 14th day of the experiment, they were removed. The lighting program was carried out via a timer with 20-W lamps, as recommended by the strain.

### Sample collection

At 43 days of age, the chickens were slaughtered by cervical dislocation, and subsequently, a fragment of the right lobe of the liver of each bird was collected using a stainless-steel knife. Each fragment was sectioned into slices, transferred to cryovials, and then stored in a freezer at -80 °C until use in proteomic assays. From the collected samples, four pools were made through homogenization and maceration of liver samples from the same group, corresponding to the four treatments studied. Samples of 10 birds per treatment were considered.

### Fractionation of broiler chickens hepatic proteome

The protein fraction extraction was carried out using 200 mg of pool of each liver sample, which were subsequently subjected to the extraction process with 1000 µL ultrapure water (18.2 MΩ cm^-1^) by means of homogenization in a cell disruptor (BEAD RUPTOR4). Subsequently, the suspensions obtained were centrifuged at 9503 × g at 4 °C for 15 min, using a refrigerated centrifuge Hettich Universal 320R. After obtaining transparent protein extracts (without the presence of impurities) was used the total precipitation strategy in ice-cold acetone to obtain of protein pellets^31^. For this, 200 µL of supernatant (hepatic tissue extract obtained after centrifugation) and 400 µL of ice-cold acetone 80% (v/v) were added to 2 mL microtubes and taken to the refrigerator where they remained at rest for 1.5 h at 4 °C. After observing protein pellet precipitates, the microtubes were centrifuged at 10,000 rpm at 4 °C for 15 min. After this process, the supernatant was discarded, and protein pellets were obtained.

The protein pellets obtained were solubilized in a specific buffer solution (7 mol L^−1^ of urea; 2 mol L^-1^ of thiourea; 2% (w/v) CHAPS (3-[(3-cholaminopropyl)-dimethylammonium]-1-propane sulfonate); 0.5% (v/v) ampholytes pH ranging from 3 to 10; and 0.002% (w/v) bromophenol blue) for performing the liver proteome fractionation process by 2D PAGE (Two-dimensional electrophoresis) according to the procedure already published in previous papers^31–36^ and briefly described to follow.

Before starting the 2D PAGE runs, the total protein determination in each protein pellet solution was performed using the Biuret method^37^. With the total protein concentration values, the concentrations in each protein pellet solution were adjusted to contain 1.50 µg µL^−1^ of total protein. Subsequently, 250 µL aliquots (this volume containing 375 µg of protein) were transferred to IEF (isoelectric focusing) strips with a pH gradient in the range of 3–10. The isoelectric focusing step was then carried out to separate the proteins by means of their respective isoelectric points – pIs, first dimension of protein fractionation process.

After completing the isoelectric focusing step, the IEF strips were subjected to a hydration process in a reducing solution and later in an alkylating solution^33^, and after this step, the IEF strips were transferred to a 12.5% (v/v) polyacrylamide gel together with a standard containing proteins of different molecular masses and submitted to the separation process in the second dimension (separation according to the molecular mass of the proteins) using the experimental conditions already described in literature^31–36^. Finished the 2D PAGE runs, the gels were carefully removed from the glass plates and immersed in a fixative solution containing 10% acetic acid (v/v) and 40% ethanol (v/v) for 30 min. After this time, the fixative solution was removed, and then colloidal Coomassie G-250 dye (USB, Cleveland, Ohio, United States) was added, remaining in contact with the gels for 72 h on a shaking table. Subsequently, the dye was removed, and the gels were washed with ultrapure water until the protein spots were completely revealed.

After complete washing, the gels were scanned, and the images were analyzed using Image Master Platinum v.7.0 software. The analysis process consists of counting the number of protein spots of each gel, percentage of correlation calculation (matching) between the repetitions of gels, and the obtaining of isoelectric point (pI) and molecular mass (MM) values of each spot^35^. All the protein fractionation process by 2D PAGE was performed in triplicate (using three gels per run of each treatment). The difference in abundance of protein spots (upregulation, downregulation, and presence/absence) between the studied groups was determined by analysis of variance (ANOVA) performed by the same software, considering a significance level of 5% (*P* < 0.05)^34^.

### Copper and manganese determinations

For the determination of copper and manganese in samples of liver tissue and protein pellets, mineralization of liver pool samples (duplicate) and protein pellets (triplicate) was performed with ultrapure concentrated sulfuric acid and 30% hydrogen peroxide (m/m) by heating in a digester block. After complete digestion (transparent extract), the acid extracts were increased to 5.00 mL in calibrated tubes for subsequent quantification of minerals by flame atomic absorption spectrometry (FAAS)^38^.

The mineral determinations in the protein spots were carried out only in the gels of groups high Cu/Mn SO_4_ and high Cu/Mn (OH)Cl. These groups were chosen due to the higher levels of supplemented Cu and Mn, thus enabling the mineral detection in the protein spots. For this, the spots were cut out of the gels and transferred to 5 mL digestion tubes, with 500 μL aliquots of ultrapure concentrated sulfuric acid and 200 μL of 30% hydrogen peroxide (m/m) being added to each tube. The set of tubes was placed in a digester block until complete mineralization of the samples (transparent extract). The acid extracts obtained were increased to 5 mL with ultrapure water (18.2 MΩ cm^−1^)^39,40^. Then, Cu and Mn were determined by graphite furnace atomic absorption spectrometry (GFAAS).

For the construction of analytical curves, copper and manganese standard solutions in 0.10 mol L^-1^ hydrochloric acid medium were prepared from the dilution of Titrisol MERK standards containing 1000 mg L^−1^ of the analytical standards. The optimal concentration ranges of the analytical curves were suggested in the equipment manual. A blank was prepared for each analytical curve. The operational conditions used in the copper and manganese determinations were those described in the equipment manufacturer's manual (Cookbook, Shimadzu AA—6800, 2000), with some modifications according to procedures described by Santos et al^41^. and Braga et al^40^. The determination of total copper and manganese concentrations in liver tissue samples and protein pellets was performed using the same procedures described above. Cu and Mn concentrations were analyzed using the ANOVA option of Minitab 17 software, and means were compared by Tukey’s test (*P* < 0.05).

### Mass spectrometry analysis

Protein spots differentially expressed in the presence of Cu and/or Mn were extracted from the gels with the aid of a scalpel, cut into segments of approximately 1 mm^3^, transferred to microtubes containing 1 mL of 5% acetic acid (v/v) and subjected to the following steps: dye removal, protein reduction and alkylation and tryptic digestion using 10 ng mL.^1^ trypsin solution. The tryptic digestion of the spots was performed using a specific commercial kit (In-GelDigestZP Kit). The peptide sequences in the extracts obtained by the tryptic digestion process were characterized by liquid chromatography tandem mass spectrometry (LC‒MS/MS). Aliquots of the eluted peptide solutions were analyzed using the nanoAcquity UPLC system coupled to the Xevo Q-TOF G2 mass spectrometer (Waters, Manchester, UK) with electrospray ionization system (Waters, UK), which was equipped with HSS T3 column (Acquity UPLC HSS T3 column 75 mm × 150 mm; 1.8 µm, Waters) and operated in positive ion mode. The data obtained were processed using Protein Lynx Global Server (PLGS) version 3.0 and the UniProt databases were used to identify proteins^34,39^.

### Bioinformatics analysis

Protein identification was performed using the UniProt database using the *Gallus gallus* genome. The FASTA sequences of the proteins were obtained from the same database and used in the functional analysis using Blast2GO v.6.0.3 software. From the GO terms (Gene Ontoloy), the program classified the protein sequences into three domains: cell component, molecular function, and biological process. The String online database (string-db.org v.11.5) was used to build the protein‒protein interaction network considering a minimum confidence score of 0.400 (medium confidence). The sources of interaction considered were experiments, databases, coexpression and cooccurrence. Through the String plugin, enrichment analysis of the KEGG pathway (Kyoto Encyclopedia of Genes and Genomes)^42,43^ was performed using Cytoscape software (v.3.9.1) to evaluate the enriched metabolic pathways (FDR < 0.05). For the analyses, only proteins with scores above 60 were considered.

### Supplementary Information

Supplementary Information.

## Data Availability

The datasets generated and analysed during the current study are available in the [UNESP Institutional Repository] repository, [http://hdl.handle.net/11449/242914].
