# Supplementary material for: Metalloproteomic analysis of liver proteins isolated from broilers fed with different sources and levels of copper and manganese
Source: Sci Rep. 2024 Feb 28;14:4883. doi: 10.1038/s41598-024-55478-8 (PMC10902370; doi:10.1038/s41598-024-55478-8)
Supplement: Supplementary file 1 — Supplementary Information. [file 41598_2024_55478_MOESM1_ESM.pdf]

Supplementary Information

Metalloproteomic approach of broilers liver fed with diet from different sources and levels of copper and manganese

Renata Aparecida Martins[a], Andrey Sávio de Almeida Assunção[a], José Cavalcante Souza Vieira[b], Leone Campos Rocha[a], Priscila Michelin Groff Urayama[a], Marília Afonso Rabelo Buzalaf[c], José Roberto Sartori[a], Pedro de Magalhães Padilha[b*]

[a]*School of Veterinary Medicine and Animal Science, São Paulo State University (UNESP), Botucatu, São Paulo, Brazil.*

[b]*Institute of Biosciences, São Paulo State University (UNESP), Botucatu, São Paulo, Brazil.*

[c]*University of São Paulo, (USP), Bauru, Brazil.*

***Corresponding author**

Pedro de Magalhães Padilha, Institute of Biosciences, São Paulo State University, Street Prof. Dr. Antonio Celso Wagner Zanin, 250, Botucatu, São Paulo, 18618-693, Brazil. E-mail: pedro.padilha@unesp.br

Table S1. Percentage of ingredients and calculated nutritional composition of the basal diet.

**Ingredients (%)**	**Pre-starter (1-7 d)**	**Starter (8-21 d)**	**Grower (22-35 d)**	**Finisher (35-42 d)**
Corn	59.06	61.58	64.44	68.75
Soybean meal, 46%	36.64	33.81	30.31	26.48
Soybean oil	0.50	1.32	2.31	2.21
Dicalcium phosphate	1.22	0.87	0.64	0.41
Limestone*	0.95	0.98	0.94	0.87
Salt	0.52	0.48	0.46	0.44
DL-Methionine, 99%	0.340	0.290	0.270	0.250
L-Lysine HCl, 99%	0.320	0.270	0.270	0.300
Premix[1]	0.200	0.180	0.160	0.120
L-Threonine 98,5%	0.120	0.090	0.080	0.080
Choline Chloride, 60%	0.072	0.063	0.058	0.038
Salinomycin	0.055	0.055	0.055	-
Phytase 500FTU	0.005	0.005	0.005	0.005
**Calculated nutritional composition**				
AME[2] (Kcal/kg)	2,964	3,050	3,150	3,200
Crude protein (%)	22.39	21.20	19.80	18.40
Calcium (%)	0.93	0.84	0.76	0.66
Available phosphorus (%)	0.47	0.40	0.35	0.31
Sodium (%)	0.23	0.21	0.20	0.20
Potassium (%)	0.87	0.83	0.77	0.71
Chlorine (%)	0.42	0.39	0.37	0.37
Lysine dig (%)	1.32	1.21	1.13	1.06
Methionine dig (%)	0.66	0.60	0.56	0.53
Met+Cys dig(%)	0.95	0.88	0.83	0.77
Threonine dig (%)	0.86	0.79	0.74	0.69

*Each level and source of supplementation of Cu and Mn were included in the basal diet through the replacement of limestone.

[1]Mineral and vitamim premix (levels/kg of diet): vitamin A, 13,500 UI; vitamin D3, 3,750 UI; vitamin E, 30 UI; vitamin K_3, 3.75 mg; vitamin B_1, 3 mg; vitamin B_2, 9 mg; pantothenic acid, 18 mg; vitamin B_6, 4.5 mg; vitamin, B_{12} 22.5 µg; niacin, 52.5 mg; folic acid, 2.25 mg; biotin, 0.15 mg; selenium, 0.375 mg; iron, 50 mg; cobalt, 1 mg; iodine, 1 mg; zinco hydroxychloride, 80 mg.

[2]Apparent metabolizable energy

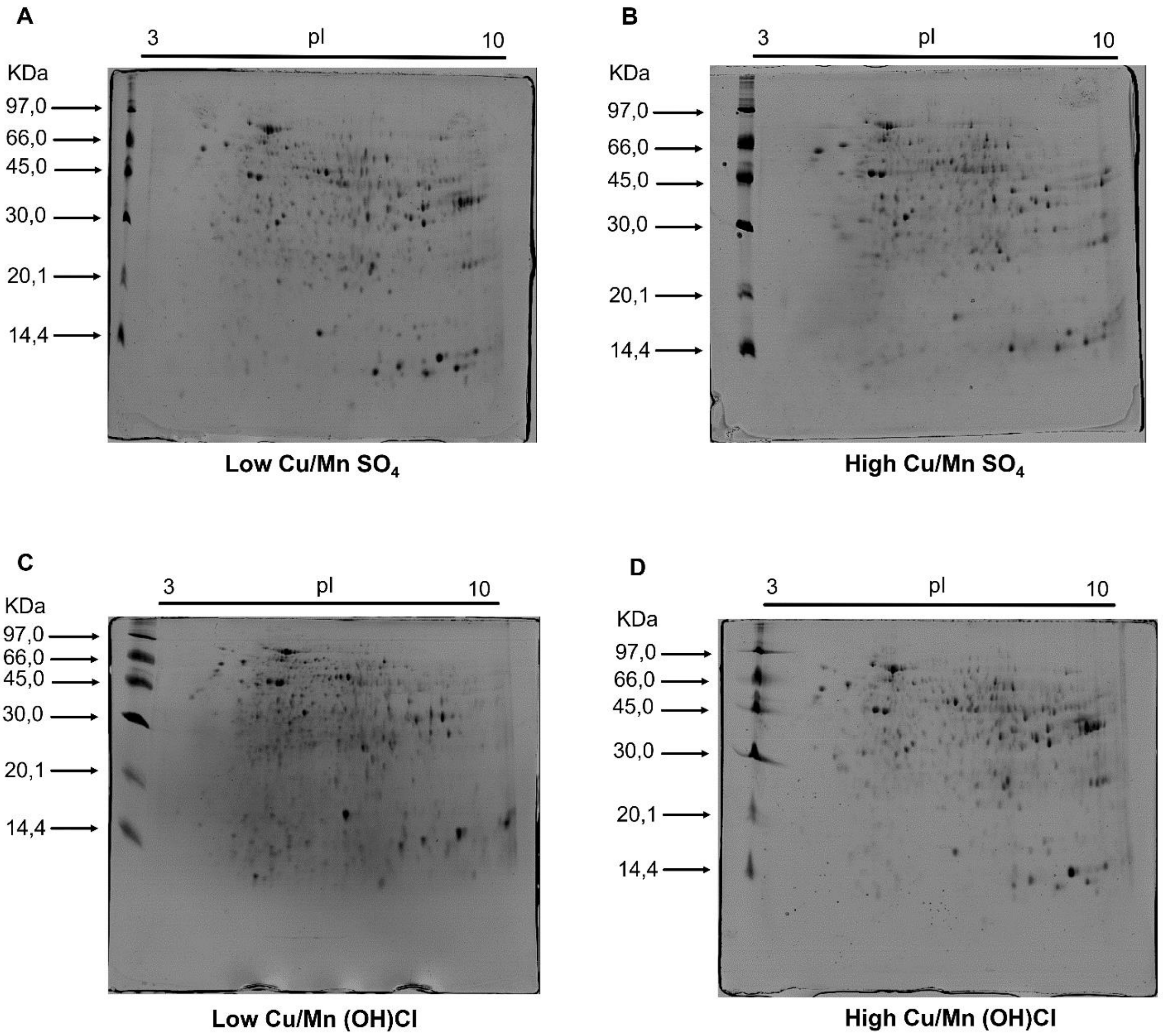

Figure S1. Representation of polyacrylamide gels obtained by 2D-PAGE from the pool of hepatic tissue samples from broiler chickens supplemented with different sources and levels of Cu and Mn. (**A**) Low Cu/Mn SO_4: 15 ppm Cu sulfate and 80 ppm Mn sulfate. (**B**) High Cu/Mn SO_4: 150 ppm Cu sulfate and 120 ppm Mn sulfate. (**C**) Low Cu/Mn (OH)Cl: 15 ppm Cu hydroxychloride and 80 ppm Mn hydroxychloride. (**D**) High Cu/Mn (OH)Cl: 150 ppm Cu hydroxychloride and 120 ppm Mn hydroxychloride.

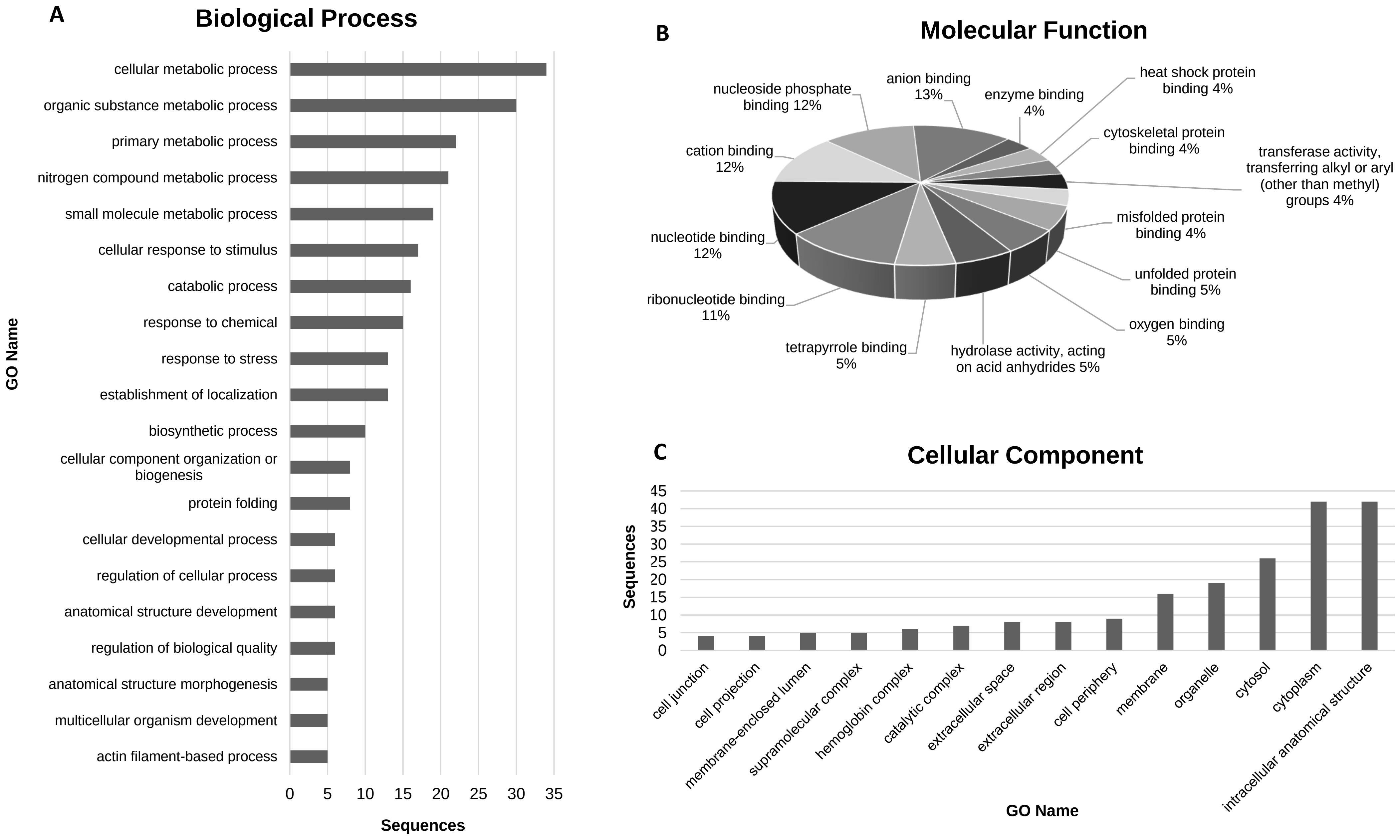

Figure S2. Functional annotation of proteins identified in protein spots associated with copper and manganese (2D-PAGE/GFAAS) using Blast2GO software. (**A**) Biological Process. (**B**) Molecular Function. (**C**) Cellular Component.
